# Congestion, decongestion, renal function and diuretics in (ESC) heart failure

**DOI:** 10.1002/ehf2.15164

**Published:** 2024-11-14

**Authors:** Jan Biegus, Piort Gajewski, Piotr Ponikowski

**Affiliations:** ^1^ Institute of Heart Diseases Wroclaw Medical University Wroclaw Poland

## Introduction

Congestion and decongestion are among the most critical pathophysiological processes in acute heart failure (AHF). Central to managing these issues is evaluating diuretic response and consistently striving to comprehend the underlying pathways that drive this response. In light of this, we have created a special virtual issue in ESC Heart Failure dedicated to exploring these topics in depth. This virtual issue serves as both a comprehensive resource of already published articles and *an invitation for authors to contribute their high‐quality research* and insights to advance the field further.

Here, we will provide some highlights of the latest papers from ESC Heart Failure dealing with the topic.

Renal function in heart failure (HF) is highly related to two major elements: neurohormonal drive and haemodynamics.^1^ The first one was further investigated in the paper by Matsumoto et al., in which associations between neuroendocrine hormones and the diuretic response to exogenous atrial natriuretic peptide (ANP) (carperitide) were examined.^2^ Lower endogenous plasma ANP levels were significantly associated with a greater diuretic response to exogenous ANP. High vasopressin was related to the poor diuretic effects of carperitide. Systolic blood pressure, estimated glomerular filtration rate (eGFR) and prior use of loop diuretics did not predict the diuretic response to exogenous ANP. In contrast, vasopressin and plasma ANP levels independently predicted the response.^2^

The relations between central haemodynamics and renal function have been analysed in the cohort of 1001 advanced HF patients from Sweden. In the study, elevated right atrial pressure (RAP) was the strongest determinant of lower measured GRF.^3^ It is important to stress that RAP was a more significant renal function determinant than mean arterial pressure or cardiac output.^3^ Of note, the neurohormonal state correlated with diuretic response and sodium and chloride homeostasis, which are known to determine both diuresis and prognosis in HF.^4^, ^5^, ^6^, ^7^ Moreover, in another study that examined 966 AHF patients with a mean age of 80 years from the KUNIUMI registry, the residual congestion [but no worsening of renal function (WRF) during hospitalization defined by an increase of serum creatinine ≥ 0.3 mg/dL] was shown to be independently associated with poor outcomes in AHF.^8^ Baudry et al. took a closer look at haemodynamic profiles (based on Forrester's classification) in non‐inotrope dependant, mostly ambulatory (79%), advanced HF patients listed for heart transplant (*n* = 837). The warm–dry, cold–dry, warm–wet and cold–wet profiles represented 27%, 18%, 27% and 28% of patients, respectively. The residual or ongoing/untreatable congestion, as depicted as a ‘wet’ haemodynamic profile, was associated with the worst outcome, irrespective of perfusion profile.^9^ These data support the notion that patients who cannot achieve optimal decongestion are at the highest risk, probably due to the highly advanced stage of the disease and underlying pathophysiology. Thus, ESC Heart Failure has also published a document dealing with diuretics in advanced HF.^10^

Going back to WRF, the other group led by Nicholas Wettersten examined the unique pre‐hospital (not in‐hospital that is usually reported) incidence of WRF and its consequences. The authors evaluated a subgroup of 406 patients from the AKINESIS study who had serum creatinine measurements available within 3 months before hospitalization and at the time of admission.^11^ Interestingly, in the cohort, one‐fourth of patients had WRF before hospitalization, which also supports the argument that the pathophysiology of the AHF starts long before the clinical presentation and hospital admission. The characteristics related to the more advanced stage of HF, like higher BNP and lower diastolic blood pressure, were significantly associated with higher odds for pre‐hospital WRF. Of note, the WRF (across all used by the authors definitions) was not associated with a higher odds of adverse in‐hospital events or a higher risk of death or HF readmission.^11^

Although diuretics are an important part of HF pharmacotherapy as they help keep the patient's fluid status in clinical control, there are several caveats related to their use. First, the overshooting diuresis, together with (recommended) fluid restriction, leads to the activation of several mechanisms to prevent hypovolaemia, starting from thirst overexcitation. In a study by van der Wal et al., one of the four stable chronic HF patients experienced severe thirst.^12^ The thirst was significantly associated with a higher dose of loop diuretics and daily urine output.^12^ Of note, excessive diuresis may also lead to hypotension or ion disturbances, which may unfavourably delay the implementation of guideline‐directed medical therapy (GDMT). Of note is that the implementation of GDMT was shown not only to prolong life and reduce the risk of recurrent HF hospitalizations but also to be associated with effective, sustainable decongestion.^13^ This is especially important as many patients have de‐escalation of the GDMT during hospitalization, which was shown to be related to worse outcomes.^14^ The study by Palin et al. observed 711 HF patients and their pharmacotherapy following hospitalizations. The ACEi/ARB dose was reduced in 21% of hospitalizations and was more common during non‐cardiovascular hospitalization. Beta‐blockers were reduced in 8% of cases. The group with ACEi/ARB reduction had worse age‐adjusted survival after discharge but no differences in HF re‐hospitalization. Although the casualty cannot be derived from this study, caution is warranted in any intervention leading to GDMT de‐escalation.^14^ On the other hand, the optimization of neurohormonal blockade has been associated with reduced requirements for diuretics. In the analysis of the MEMS‐HF population (*n* = 239), in which pharmacotherapy was guided by remote pulmonary artery pressure monitoring, sacubitril/valsartan use was associated with significantly lower utilization of loop diuretics.^15^

Lastly, the diuretic dose has been shown to be unfavourably related to outcomes in HF. In the cohort of (*n* = 700) HF patients with ambulatory, advanced HF awaiting heart transplantation, the dose of loop diuretics positively correlated with several markers of disease severity, like NTproBNP, serum creatinine/eGFR, RAP, pulmonary capillary wedge pressure and pulmonary pressures.^16^ Moreover, the ‘high‐dose’ loop diuretic group (>250 mg/day of furosemide or equivalent) was associated with increased waitlist mortality or urgent HT and a six‐fold higher risk of waitlist death in comparison to the ‘low‐dose’ group (≤40 mg/day).^16^ On the other hand, the Japanese Kyoto Congestive Heart Failure registry examined 3665 consecutive discharged home AHF patients. Out of the study cohort, 1906 (52%) patients had not received diuretics before admission, while the rest, 1759 (48%) patients, had been on diuretics before admission.^17^ Importantly, in‐hospital initiation and ambulatory continuation of loop diuretics were not associated with lower post‐discharge mortality.^17^ Changes in loop diuretic dosage at discharge did not lead to a lower mortality risk after discharge compared to situations where the dosage remained unchanged. Moreover, when comparing patients who did not receive any loop diuretics at discharge, those who were given a dose ≥ 80 mg were associated with a higher risk of post‐discharge mortality..^17^

Other authors have examined the concept of time‐to‐ (the first) diuretic dose as a rescue therapy in an AHF event. The concept assumes that the shorter the time to the first dose of diuretics, the faster the patients receive congestion relief therapy, which should translate into better outcomes. The study population consisted of 15 078 patients from seven world regions from the REPORT‐HF registry.^18^ Interestingly, the median time‐to‐diuretics was 67 min. Patients with more signs and symptoms of HF, women, and patients from Eastern Europe or Southeast Asia had shorter time‐to‐diuretics. There was no significant association between time‐to‐diuretics and in‐hospital mortality.^18^ On the other hand, it is important to remember that in selected patients, the mechanical elimination of excess fluid, such as ultrafiltration, may be a viable option.^19^

The other study explored the outcomes of ambulatory treatment for worsening heart failure (WHF) using intravenous diuretics as a preferable alternative to hospital admission.^20^ The authors retrospectively evaluated 259 WHF patients, irrespectively from the left ventricle ejection fraction. The outpatient treatment of patients with WHF was a safe approach that deserves further examination, but the efficacy of this strategy needs to be further examined in prospective randomized clinical trials.^20^

Although we did not cover all the aspects of congestion, decongestion and diuretics in (ESC) heart failure, we would like to take this opportunity to invite the authors again to submit their high‐quality research on congestion/decongestion in HF to the virtual issue of ESC Heart Failure.
